# Using nested discretization for a detailed yet computationally efficient simulation of local hydrology in a distributed hydrologic model

**DOI:** 10.1038/s41598-018-24122-7

**Published:** 2018-04-10

**Authors:** Dongdong Wang, Yanlan Liu, Mukesh Kumar

**Affiliations:** Duke University, Nicholas School of the Environment, Durham, 27708 USA

## Abstract

Fully distributed hydrologic models are often used to simulate hydrologic states at fine spatio-temporal resolutions. However, simulations based on these models may become computationally expensive, constraining their applications to smaller domains. This study demonstrates that a nested-discretization based modeling strategy can be used to improve the efficiency of distributed hydrologic simulations, especially for applications where fine resolution estimates of hydrologic states are of the focus only within a part of a watershed. To this end, we consider two applications where the goal is to capture the groundwater dynamics within a defined target area. Our results show that at the target locations, a nested simulation is able to competently replicate the estimates of groundwater table as obtained from the fine simulation, while yielding significant computational savings. The results highlight the potential of using nested discretization for a detailed yet computationally efficient estimation of hydrologic states in part of the model domain.

## Introduction

Physically-based, fully distributed hydrologic models simulate multiple states and fluxes in space and time^1–8^. These models account for the heterogeneities in hydrogeologic parameters and meteorological forcings, and have been demonstrated to enhance understanding and prediction of hydrologic processes^9–16^. However, simulations based on these models are often computationally expensive. The computation time may become prohibitively large for fine spatio-temporal resolution simulations in large domains, rendering them intractable or at least not suitable for near real-time predictions on a serial computer. To alleviate this problem, several solutions have been proposed. For example, Bhatt *et al*.^17^ tightly coupled a Geographic Information System (GIS) with a hydrologic model using a shared data model paradigm^18^ to reduce model setup time. Parallelization of distributed hydrological models^3,5,19–23^ have been performed to reduce simulation time for basin scale simulations. Many other studies have used preconditioning to accelerate serial distributed hydrologic model codes. For example, Maxwell^24^ formulated an analytical Jacobian as a preconditioner to speed up model simulation time. Similarly, HydroGeoSphere model employed an incomplete-LU preconditioner with bi-conjugate accelerator to reduce the time of convergence^22^.

This study implements a nested-discretization based modeling strategy that can be used for efficient distributed hydrologic model simulations, especially for applications where in addition to streamflow estimates at the watershed outlet fine resolution estimates of hydrologic states are desired only within a small part of the model domain. Examples of such problem include validation of groundwater dynamics at isolated well locations, mapping of groundwater distribution within wetlands, and characterization of interactions between a hydrographic feature (e.g. a wetland or a river reach) and its neighboring aquifer. An underlying commonality in these problems is the need for fine scale delineation of hydrologic states or fluxes at least in part of the model domain (hereafter referred as the target area). Notably, these problems often also require simultaneous simulation of the rest of the watershed so as to: (a) capture the flux contributions from rest of the model domain into the target area. For example, in order to capture groundwater dynamics in a wetland that is adjacent to a stream, one has to estimate the stage in the neighboring stream as well. This stage is in turn dependent on runoff and groundwater contributions to streamflow in the upstream region of the target area; and (b) provide estimates of additional states and fluxes. For example, the modeling goal may also include simulating streamflow at the watershed outlet.

**Figure 1:**
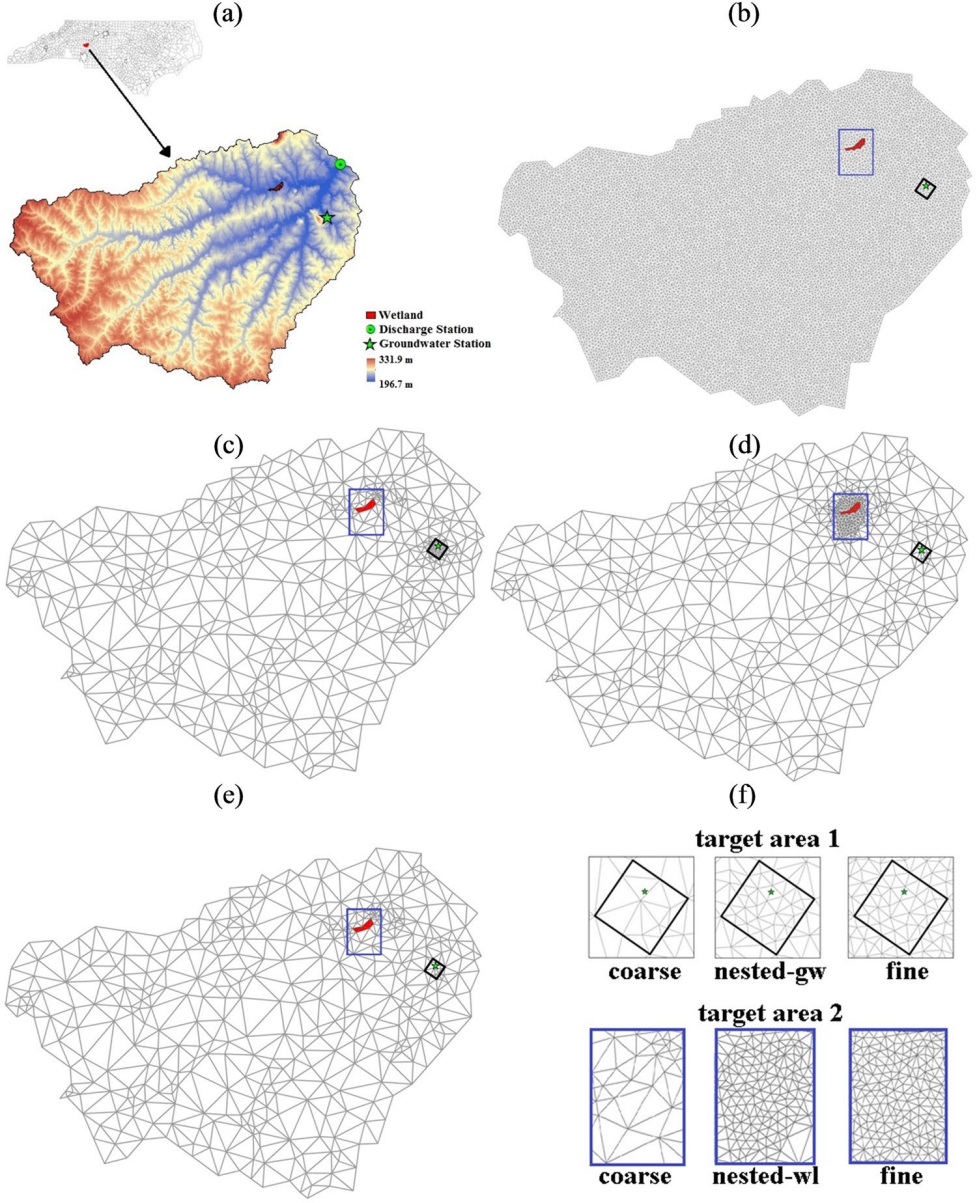
Second Creek watershed (**a**) and the four considered discretization configurations i.e., fine (**b**), nested-gw (**c**), nested-wl (**d**), and coarse (**e**). Nested-gw and nested-wl has nested discretizations around groundwater well and wetland, respectively. Coarse, nested, and fine discretizations in and around groundwater station (target area 1) and wetland (target area 2) are illustrated in (**f**). The watershed map and discretizations were generated using the PIHMgis v 3.0 (http://www.pihm.psu.edu/pihmgis_downloads.html).

The working hypothesis of the study was that although a watershed-wide or globally fine mesh provides a spatially resolved simulation result everywhere in the watershed, a locally refined grid could ensure similar estimates of streamflow at the watershed outlet and hydrologic states within the target region while ensuring significant savings in computation time. To evaluate this hypothesis, we considered two test applications. The first application dealt with the need to model groundwater water table (GWT) dynamics at the well location. The second application’s goal was to map the wet-regions (or areas with GWT within a certain threshold distance from the land surface) in a wetland as well as to evaluate the flux exchange between the wetland and adjacent stream and aquifer. Four hydrologic simulations with markedly different mesh configurations viz. a globally fine discretization, a nested discretization around groundwater well (called nested-gw from this point onwards), a nested discretization around wetland (called nested-wl from this point onwards), and a coarse discretization, were performed (Fig. 1). Accuracies and computation time of these four simulations were compared to evaluate the efficacy and efficiency of nested-discretization for simulating local hydrology within a given target area.

## Results and Analysis

### Computation Cost

**Figure 2:**
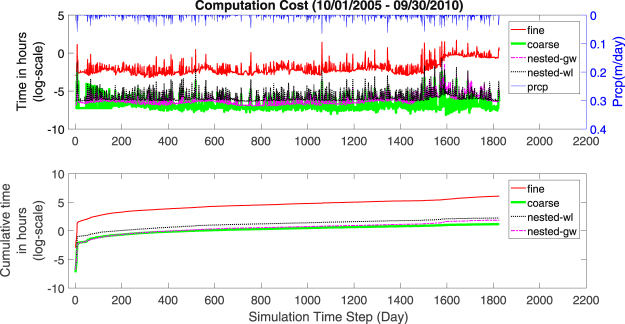
Computation time per one minute simulation step (above) and cumulative computation time (below) for fine, nested-wl, nested-gw, and coarse simulations. Prcp denotes the precipitation time series. Log scale on the y-axis has *e* (=2.718) as its base.

The four simulations were performed on a dedicated Intel Xeon E5-2680 V3 2.5 GHz processor with 256 G RAM. The simulation time was clocked after each 1 minute advance of the simulation (Fig. 2). Expectedly, the result indicated that the time it took to advance a minute of simulation varied over the simulation period. The computation time was longer after precipitation occurrences as these events triggered fast surface overland flow, infiltration, and recharge, and sharp changes in interactions between surface and subsurface flow. To accurately capture the rapid changes in states and fluxes, the solver adaptively refines the modeling time steps resulting in an increase of computation time.

The five-year simulations cost 435.28 h, 6.50 h, 9.46 h, and 3.24 h for fine, nested-gw, nested-wl, and coarse simulations, respectively (Fig. 2). This reveals that nested-gw and coarse simulation took only 1.4% and 0.7% of the computation time as fine simulation for the five year simulation period. Even the nested-wl simulation took just 2.2% of the time. In the wet year (2010) and the dry year (2007), the coarse simulation yielded computational savings of 99.7% and 98.9% respectively. For, nested-gw and nested-wl, the corresponding savings were 98.7% and 98.6%, and 98.7% and 97.2% respectively. These results show that grid coarsening leads to significant reduction in computation time. Higher gains were obtained in wet years because the increase in simulation cost for the wet year w.r.t. the average year was much higher for the fine discretization.

### Intercomparison of hydrologic states and fluxes

Given that nested simulations were found to yield significant computational savings, our next goal was to evaluate its effectiveness in capturing states and fluxes at defined locations. In this regard, we first performed comparisons of streamflow estimates at the watershed outlet. This was followed by comparison of relevant groundwater states and fluxes at the two selected target areas.

#### Streamflow at the gauging location

**Figure 3:**
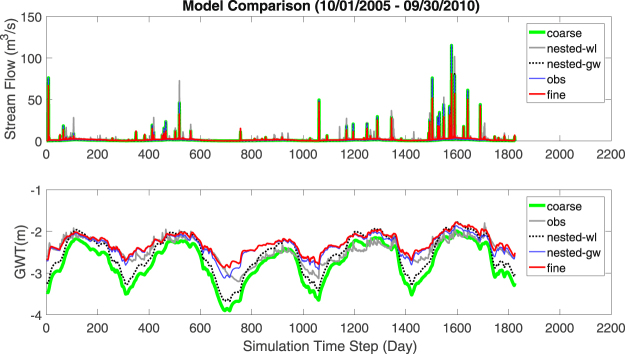
Modeled streamflow and groundwater at the gauging location from fine, nested-wl, nested-gw, and coarse simulations. Semi-log version of the hydrograph plot (top) is shown in Supplementary Fig. S1.

Figure 3 shows the hydrographs of simulated and observed streamflow at the gauging station. Modeled streamflows from the four mesh configurations show very similar performance to each other, although there were some differences. For example, coarse and nested simulations generally over-predicted high flows w.r.t. the fine simulation. Over five years, with respect to the fine simulation, the Nash-Sutcliffe efficiencies (NSEs) of daily streamflow for nested-gw, nested-wl, and coarse simulations were 0.823, 0.837, and 0.823 respectively. With respect to the observed data, NSEs of modeled daily streamflow were 0.677. 0.630, 0.645, and 0.630 for fine, nested-gw, nested-wl, and coarse simulations, respectively. These results indicate that almost identical performance in streamflow simulations can be achieved from meshes of significantly different resolution. This is because streamflow is an integrated hydrologic response of a watershed. As long as a model resolution is fine enough to capture the overall spatial extent and distribution of primary controls on runoff and recharge such as the topography, hydrogeology, and land cover, similar streamflow series is expected^25,26^.

#### Groundwater table at the well location (target area 1)

In contrast to the high NSE of 0.832 for streamflow in the coarse simulation w.r.t. the fine simulation, the NSE of GWT depth time series was only −3.698. After local refinement i.e., using nested-gw discretization, the NSE improved to 0.891. For the bias corrected GWT series i.e., after removal of the respective mean value from each GWT time series, NSEs for the coarse and nested simulations were −0.040 and 0.959, respectively. Notably, NSE for nested simulation was again significantly better. The result implies that mesh resolution near the groundwater well location plays a significant role on the accuracy with which groundwater table dynamics can be simulated. Even when the errors due to process representation and forcing data are negligible, a user interested in validating a distributed hydrologic model at the groundwater well is likely to obtain an incorrect estimate if a coarse discretization is used. This may force the user to consider incorrect or alternate parameter configurations to compensate for errors in groundwater estimate at the validation site. In contrast, nested simulation is able to capture the estimates of groundwater table and streamflow at the gauging locations, while yielding significant computational savings.

#### Groundwater dynamics within the wetland (target area 2)

Given that groundwater table height and its spatial distribution impact many functions of wetland, such as methane release, nitrification, or carbon storage, here we intercompared three variables viz. average GWT (*GWT*_*avg*_), groundwater table distribution, and wet area fraction (*WAF*) across the different discretizations.

##### Average groundwater table:

**Figure 4:**
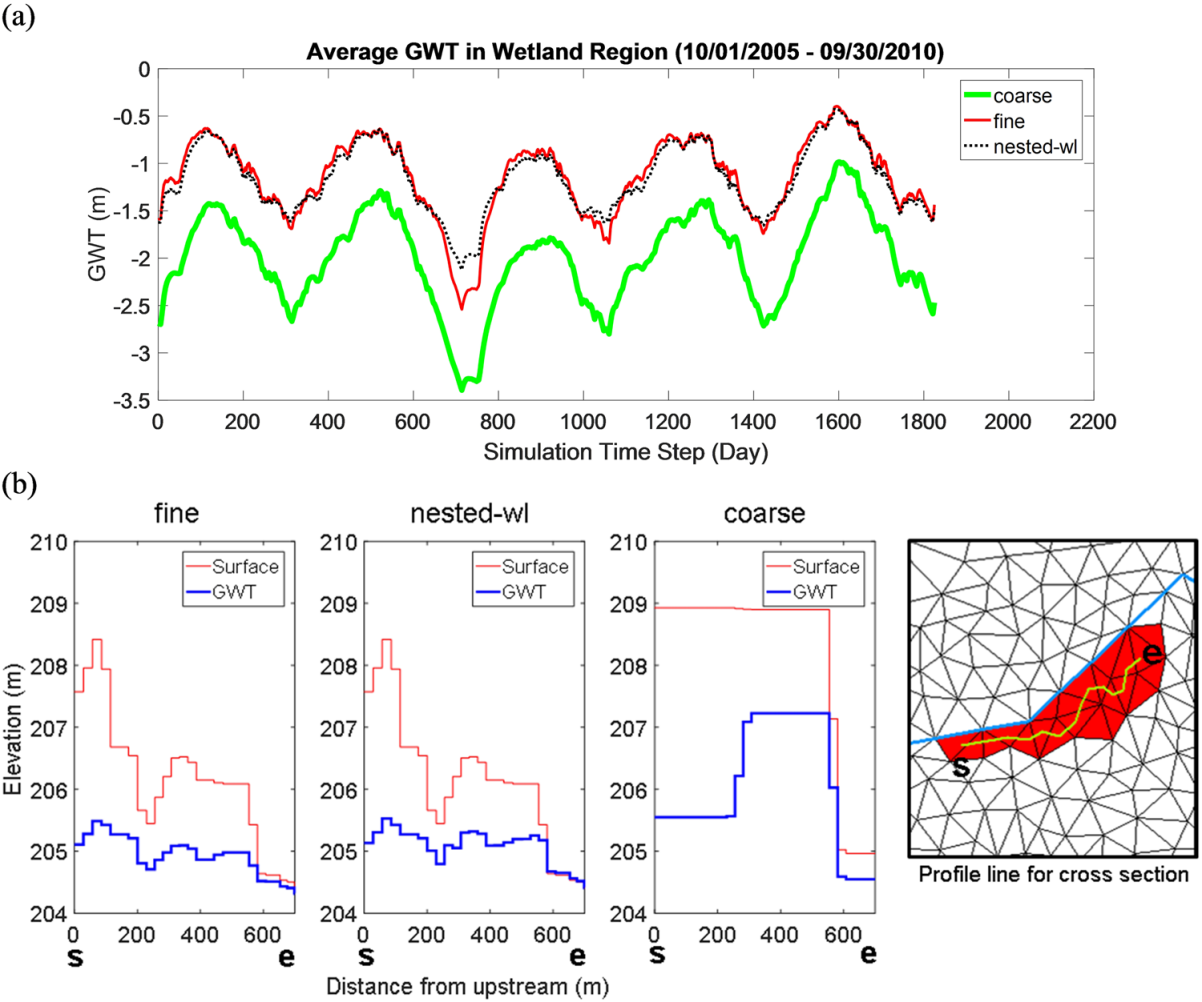
Model intercomparison of GWT estimates within the target wetland: (**a**) spatially averaged GWT variability, and (**b**) Surface topography and GWT profiles along a profile section within the wetland. The profiles were generated at the 17^th^ hour on the 520^th^ day. The profile line starts at ***s*** on the left and ends at ***e*** on the right.

**Figure 5:**
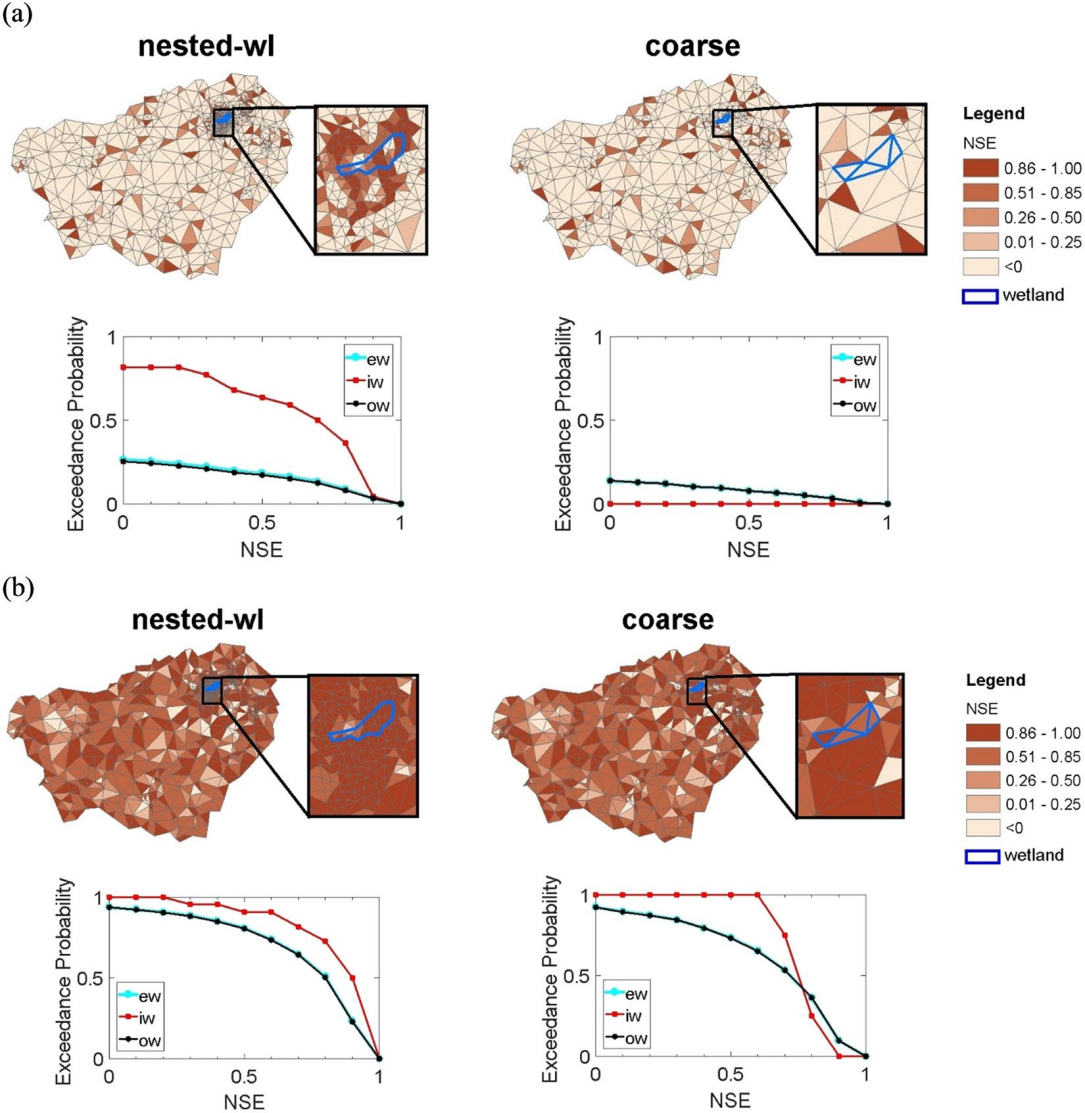
Comparison of model fit with respect to fine simulation over the 5 years simulation period. Comparison was performed using the simulated groundwater table time series (**a**) and mean removed groundwater time series. Here, ew denotes “entire watershed”, iw denotes “inside of wetland”, and ow denotes “outside of wetland”.

Temporal dynamics of *GWT*_*avg*_ in nested-wl and fine simulations closely resembled each other (Fig. 4(a)). In contrast, *GWT*_*avg*_ in coarse grid simulation was lower. The mean of *GWT*_*avg*_ over five years was −1.15 m, −1.16 m, and −2.01 m for fine, nested-wl, and coarse simulations, respectively. Nested-wl and coarse simulations yielded NSEs of 0.94 and −3.34 w.r.t. the fine case. After removal of respective means, the NSE for coarse simulation improved to 0.88. These results highlight that with respect to the fine simulation, there was a significant bias in *GWT*_*avg*_ obtained from the coarse simulation. However, the coarse simulation reasonably captured the temporal variation of *GWT*_*avg*_ within the wetland. Notably, satisfactory performance of the coarse simulation for bias-removed GWT, as observed in this case, is not expected everywhere in the watershed (Fig. 5(b)). In fact, NSE of bias-removed GWT at the groundwater observation gauge (in Fig. 3) was only −0.040.

##### Groundwater table distribution:

Considering GWT estimated from the fine simulation as a reference, 64% of the discretization elements within the target wetland in the nested-wl case showed NSE greater than 0.5. The fraction decreased to 36% for NSE greater than 0.8 (Fig. 5). Corresponding fractions for the rest of the watershed were 17% and 8% respectively. Notably the performance within the wetland for nested discretization was much better than outside of it. For the coarse discretization, none of elements had NSE greater than 0 within the wetland. The difference in GWT within the wetland between coarse simulation and the other two cases (nested-wl and fine) is largely due to the disparities in topographic representation. For example, in the considered case, coarsening increased the topographic elevation of the wetland and altered its heterogeneity thus influencing flow interactions with its neighbors and consequently the GWT distribution (Fig. 4(b)).

As was the case for target area 1, after removal of respective groundwater means, the NSE improved. For nested-wl case, fraction of elements with NSE larger than 0.5 and 0.8 increased to 91% and 73%, respectively. Corresponding numbers for the coarse simulation were 100% and 25%. Modest performance of the coarse simulation points to its suitability for capturing the groundwater table variations within a wetland, even though the GWT magnitude is biased.

##### Wet Area Fraction:

**Figure 6:**
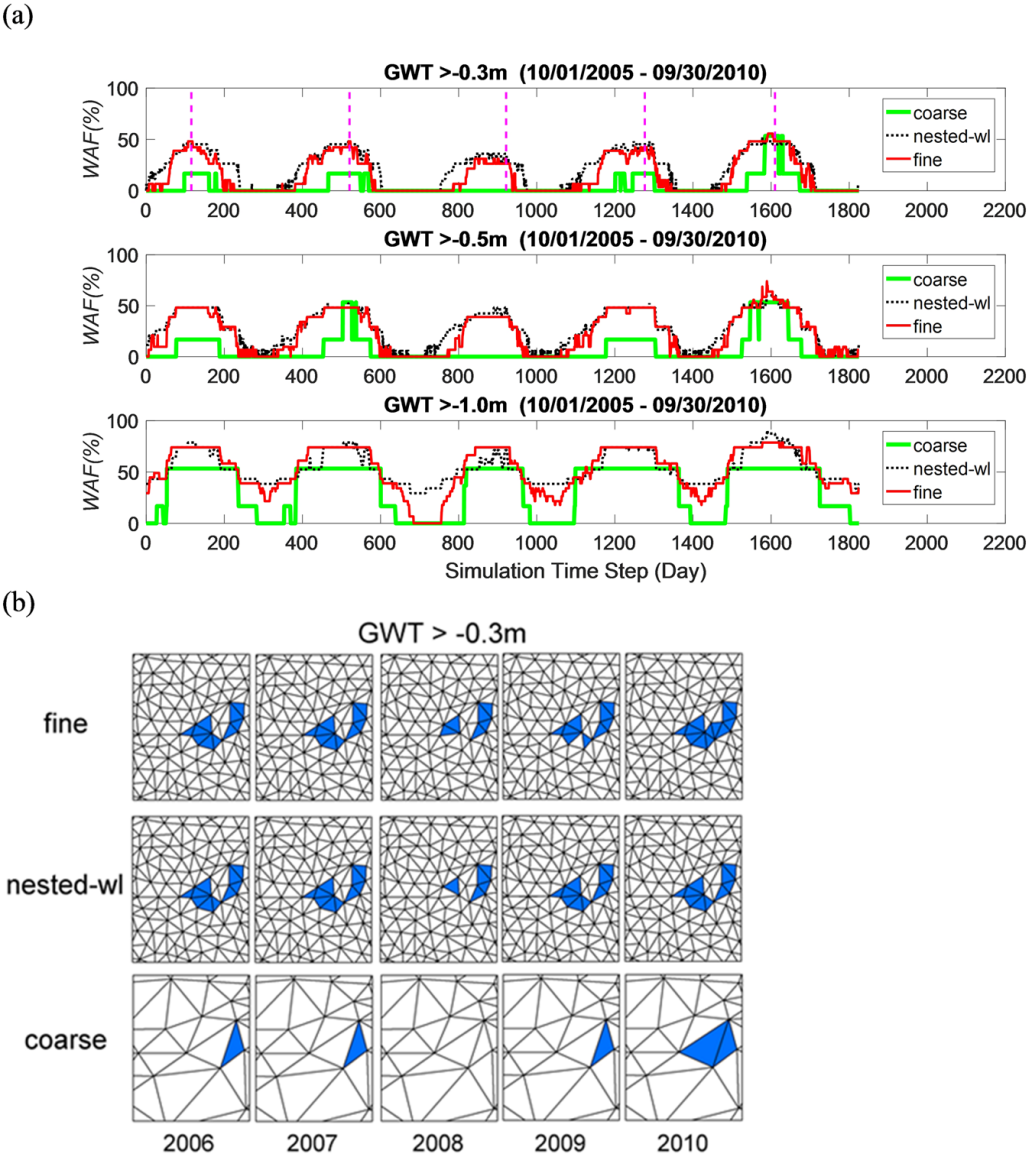
Comparison of modeled *WAF* under three groundwater depth thresholds (**a**) and spatial distribution of wet elements (**b**) for GWT > −0.3 m at the times identified by vertical hatched lines in the topmost panel of (**a**).

Over the five years, mean *WAF* for groundwater table depth threshold value of −0.3 m, −0.5 m and −1.0 m were 15.8%, 23.7%, and 52.8% for the fine simulation, 21.3%, 27.3%, and 53.9% for nested-wl simulation, and 4.2%, 7.1%, and 33.1% for coarse simulation (Fig. 6). The NSEs for the three thresholds were 0.72, 0.87, and 0.74 for nested-wl simulation and 0.01, −0.23, and −0.15 for coarse simulation. Spatial distribution of wet locations i.e., areas with GWT higher than a prescribed threshold, also showed higher degree of similarity between nested-wl and fine grids (Fig. 6b). These results highlight that nested-wl and fine simulations show similar wet period dynamics in wetlands, both spatially and temporally. As wet period dynamics play an important role on ecosystem services provided by wetlands^27^, choice of gridding strategy is likely to influence the ability of models for simulating these services.

#### Flux dynamics at the wetland boundary

Groundwater dynamics within the wetland is strongly influenced by its interactions with neighbors. Here we evaluated the ability of coarse and nested discretizations to capture the flux interaction between the wetland and its neighbors.

##### Wetland-aquifer interactions:

**Figure 7:**
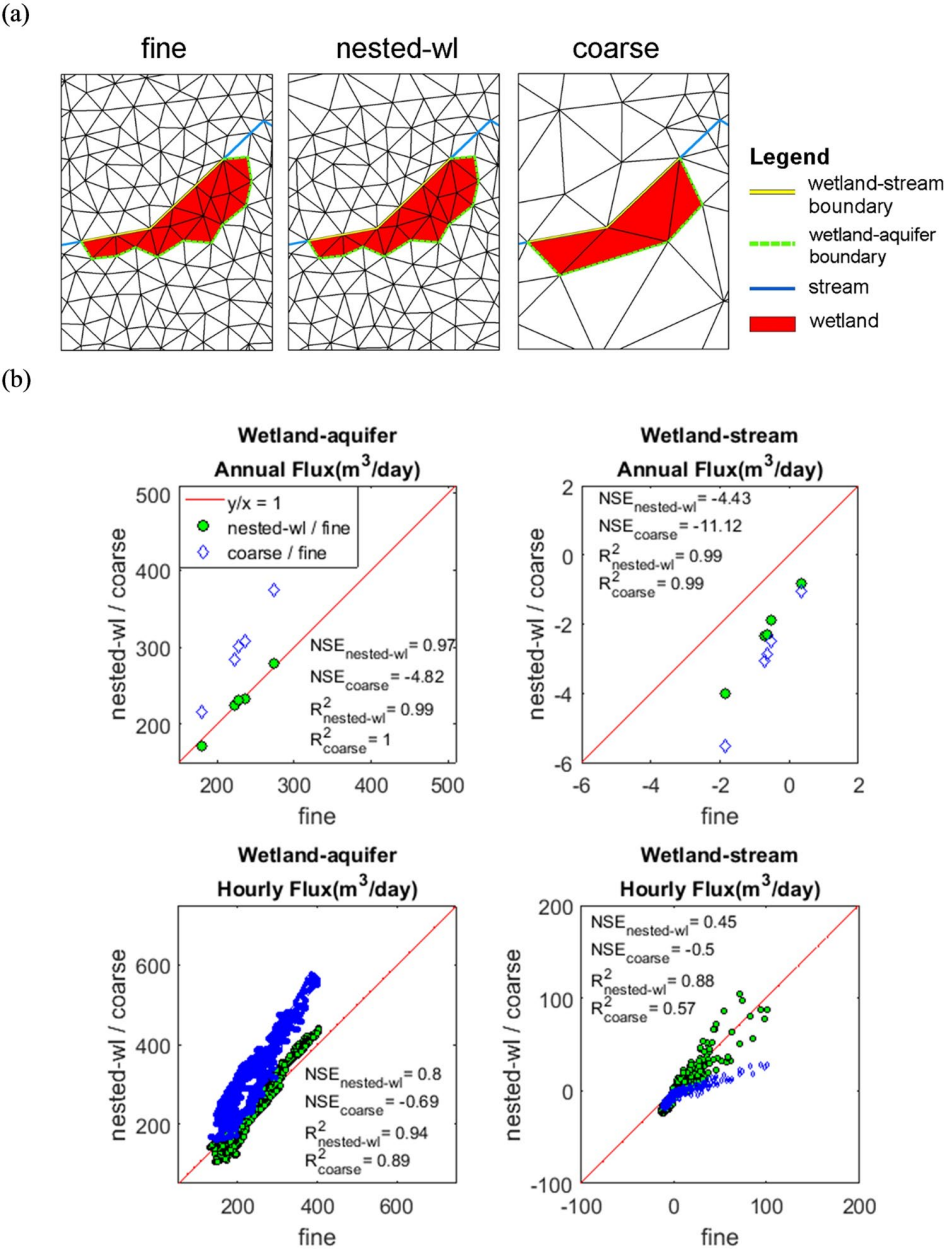
Comparison of wetland-stream and wetland-aquifer interaction fluxes between the three discretizations. Fine simulation is used as reference model. The interaction boundaries are illustrated in (**a**).

Subsurface lateral flux was calculated along the wetland-aquifer boundary identified in Fig. 7(a). The result shows a close agreement between nested-wl and fine simulation with NSE of 0.97 at annual resolution and NSE of 0.80 at hourly resolution (Fig. 7(b)). Corresponding NSEs between coarse and fine simulation were −4.82 and −0.69, respectively. Over the five simulation years, nested-wl simulation yielded only 0.2% more outflow than the fine simulation, while the bias for the coarse simulation was around 30%. Larger net input flux to the wetland in coarse simulation can be attributed to larger groundwater contributing area, which in turn is due to the difference in topographic representation outside the wetland. For coarse discretization, the time-averaged groundwater contributing area was 3.79 km^2^ whereas it was only 2.62 km^2^ and 2.54 km^2^ for nested-wl and fine discretizations. This led to larger groundwater recharge in the contributing area of the coarse discretization resulting in larger lateral groundwater flux to the wetland.

##### Wetland-stream interactions:

**Figure 8:**
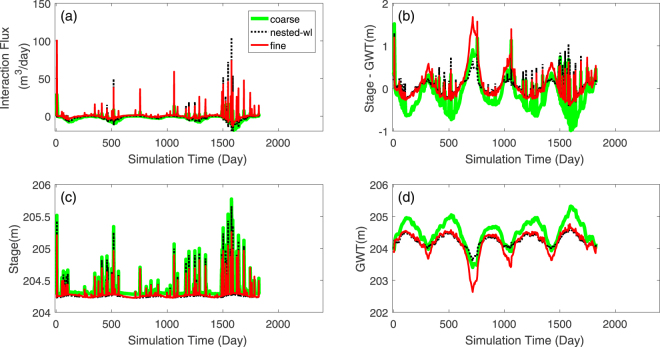
Plots of wetland-stream interaction flux (**a**), difference between river stage and GWT (**b**), river stage (**c**), and GWT time series (**d**). Negative Stage-GWT implies that stage is lower than GWT and wetland discharges into the stream. A positive Stage-GWT implies wetland recharges from stream.

Wetland-stream interaction flux was evaluated along the wetland-stream boundary identified in Fig. 7(a). Since upstream contributing area of the stream-reach neighboring the wetland is very similar in the nested-wl and coarse discretizations, it is expected that the streamflow magnitude (and the stream stage) in the stream reach just upstream of the wetland are almost identical for the two discretizations (Supplementary Fig. S2). However, differences in discretization of the stream reach adjacent to the wetland in coarse and nested-wl simulations is likely to introduce some differences in stage elevation (S) as well. Figure 8(c) shows that during high flow periods (i.e., during and immediately after precipitation events), the stage, S, in the stream for coarse and nested-wl simulations were very similar. However, during the dry periods, stage in nested-wl was higher than in coarse simulation as the stream bed elevation was higher in nested-wl discretization. Since the upstream contributing area representation in fine discretization was very different than in coarse and nested-wl discretizations, stage for fine discretization was expectedly different than in other two cases (Fig. 8(c)). In regards to the average GWT in the wetland cells that are adjacent to the stream, GWT in fine and nested-wl simulations were similar, at least during the groundwater recharge periods (Fig. 8(d)). However, the GWT in coarse simulation showed large differences during both wet and dry periods. Because of these reasons, the interaction flux between wetland and the streamflow reach, as obtained by nested-wl and coarse simulations, did not show a good agreement with that obtained from the fine simulation. For example, over the five simulation years, annual estimates from nested-wl simulation yielded an NSE of −4.4 with respect to the fine simulation (Fig. 7(b)). The corresponding NSE for coarse simulation was also poor (NSE = −11.1). These results show that at annual scale, both nested-wl and coarse simulations yield less net water loss to stream. At an hourly scale (Fig. 7(b)), the interaction flux between wetland and stream were both positive (wetland gains water from stream) and negative (wetland loses water to the stream). In response to precipitation events, nested-wl sometimes over-predicted and at other times under-predicted wetland-stream interactions. In contrast, coarse simulation mostly under-predicted w.r.t. the fine simulations in response to events. The over- and under-prediction can be explained based on the difference between river stage (S) and GWT elevation adjacent to the river (Fig. 8). During high flow events when nested-wl over-predicted wetland-stream flux, it was generally because of higher S minus GWT (S-GWT) than fine simulation which in turn was due to a higher S in nested-wl than in fine simulation. S-GWT for the coarse simulation was almost always smaller than the fine case as GWT in coarse simulation was higher. During low flow periods, nested-wl and coarse simulations yielded smaller difference (Fig. 8(a)). Although the disparity in S-GWT during dry period was large (Fig. 8(b)), since the water depth in the stream was close to zero, the interaction flux was very small as well. The results show that the nested simulation may not accurately capture the boundary fluxes as simulated by the fine simulation, if these fluxes are heavily influenced by discretization outside the target region. Notably, in this case the wetland-stream flux was two orders of magnitude smaller than wetland-aquifer flux. Hence, the differences in wetland-stream flux between the three simulations did not affect the groundwater dynamics in the wetland as much.

## Discussion

Fully distributed integrated hydrologic models have the advantage of simulating multiple states and fluxes at fine spatio-temporal resolution. However, fine model resolution significantly increases the computation cost. This study demonstrates that a nested discretization strategy can potentially alleviate this problem, especially for applications where fine resolution estimates of hydrologic states are desired only within a small part of a watershed. Our results show that nested simulations can yield similar estimates of groundwater in the target region and streamflow at the watershed outlet as that provided by the fine simulation, but at a fraction of computation cost. For example, nested-wl simulation saved 97.8% computation time compared to the fine simulation over a five year simulation period. We also find that the attained computation savings are dependent on the meteorological forcing regime. For instance, in the wet year, nested-wl simulation saved 98.7% time cost of fine simulation while the percentage reduced to 97.2% for the dry year. This indicates that nested modeling is expected to provide higher performance yields in wetter settings.

The study’s demonstration of the ability of nested simulations to simultaneously capture streamflow at the watershed outlet and groundwater table at the gauging station has wide-ranging ramifications, especially for validation and evaluation of distributed hydrologic models. Nested discretization allows model evaluation against both surface water (/streamflow at the outlet) and groundwater states, which is important for reducing uncertainty in estimates of water budget partitioning^28^, without the need for fine discretization everywhere in the watershed. In contrast, a user interested in evaluating a distributed hydrologic model at the groundwater well is likely to obtain an incorrect estimate if a coarse discretization is used. This in turn may force the user to consider incorrect or alternate parameter configurations to compensate for errors in groundwater estimate at the validation site. This is likely to be the case for land surface models (e.g., Noah-MP^29,30^, VIC^31,32^, etc.) that are often applied at scales coarser than 1 km × 1 km. The study highlights that nested discretization can be an effective way of addressing this challenge. Success of the nested model in capturing groundwater dynamics in wetlands and wetland-aquifer interactions further highlights its potential for simulating a range of wetland processes and functions such as methane emissions^33^, evapotranspirative feedback^34^ to the atmosphere, and nitrate removal^35^, that are closely related to wetland groundwater and inundation dynamics.

In addition to capturing more accurate spatio-temporal distribution of groundwater table, our ancillary analysis confimed that nested discretizations may lead to better estimate of soil moisture and evapotranspiration as well (Figs S3 and S4). The nested discretization strategy may potentially be used in many other hydrologic applications where fine scale delineation of hydrologic states or fluxes are desired in part of the model domain. These include: (a) mapping of flood inundation areas, contaminant plume extent etc.; (b) quantification of localized impact of melt recharge (/transpiration) from a snow drift (/tree stand) on groundwater table (GWT); and (c) delineation of the temporal evolution of cone of depression due to groundwater pumping in a large watershed. Notably, as the physical controls on hydrologic states and fluxes to be estimated through nested modeling may vary within a watershed and from one region to next, the improvement achieved through nested discretization may vary as well. The extent of gain in computation efficiency through nested discretization is also expected to be influenced by grid resolution.

To achieve maximum computational efficiency for a given accuracy, optimal nested mesh configurations, both in terms of its resolution and spatial extent, should be derived. One of the primary reasons why nested modeling provides similar estimate of hydrologic states as the fine simulation is because of better representation of watershed properties in the target area. However, similar estimates within the target area are possible only if the net lateral fluxes from the rest of the domain into the target area are not much affected by the use of a coarse discretization outside the nested region. This may be facilitated by: (a) using a large nested region (much larger than the target area). However, this will likely lead to increase in computation load; (b) using nested discretization in a flatter terrain where lateral flow interactions between the target area and rest of the watershed is negligible; and (c) forcing the nested region boundary to align with surface water and/or groundwater divide. For example, one can use a nested discretization that extends to a topographic sub-watershed boundary. Since topographic boundaries also act as surface water divide, nested discretizations whose boundary align with topographic divide will produce overland flow estimates that are identical to one obtained using the fine scale simulation. Notably, defining such a boundary for other hydrologic states is challenging. For example, groundwater flow divide is oftentimes dynamic and may not coincide with surface water divide. To reduce the uncertainty, it is still a good option to perform nested discretization by following ridges. In circumstances where a priori knowledge needed to delineate the extent of nested region is unclear, an agglomerative scheme may be used. This may first entail delineating a nested domain that is just a little larger than the target area, and performing simulations. Additional simulations with larger and larger extent of the nested region should then be performed. Once the simulation results within the target area stops changing, users can select that nested discretization. Another option is to compare the results of a nested simulation to results of a short (in time domain) fine scale simulation. Comparisons may help evaluate the sufficiency of the nested discretization for capturing the dynamics of a hydrologic state. These two approaches may also be used for identifying the optimal resolution of the mesh, both inside and outside of the target region. A user can balance the needs of computational saving and accuracy to arrive at the decision. Derivation of the optimal mesh is expected to be especially useful in applications where the nested model has to be used repetitively. For these applications, the effort needed for optimizing the mesh configuration could be compensated by computational savings obtained through recurrent simulations.

The nested discretization strategy used here can be easily employed in other unstructured grid based fully distributed models such as InHM^1^, HydroGeoSphere^36^, and FEFLOW^37^. Modifications in the solver and use of advanced structured grid discretization softwares may also extend its applicability to structured grid based models, such as MODFLOW^38^, ParFlow^3^, and PAWS^39^. Future studies may focus on identifying regions where groundwater divide is likely to coincide with surface water divide. This could make it much easier to define the extent of nested regions, especially for modeling groundwater dynamics within a target area. Identifying watershed properties and meteorological conditions where nested discretization is expected to yield large savings will further facilitate its usage and effectiveness. Further confidence in the strategy can be gained by applying it in diverse hydroclimatic settings, and within different modeling schemes.

## Methods

### Study site

The study was conducted in the Second Creek watershed (part of South Yadkin river basin), located in southwest North Carolina (Fig. 1). The watershed (area = 325 km^2^) drains into Second Creek, in which streamflow has been measured (USGS streamflow gauge # 02120780; 35.6°N, 80.7°W) for more than thirty years. It also has a USGS groundwater gauging station (#354057080362601) where daily groundwater level has been measured since 1989. The watershed was selected because it contains multiple forested freshwater wetlands within its boundary. Physiography of the watershed is characterized by valleys and ridges oriented along the southwest-northeast direction. Watershed elevation ranges from 197 to 331 m (Fig. 1). Land cover in the watershed mainly consists of hay/pasture (37.6%), deciduous forest (32.9%), developed area (6.8%), and evergreen forest (5.4%). The most common soil types in the watershed are loam in the riverbed and riparian regions and sandy clay loam in uplands. The watershed falls in warm temperate climate with humid and warm summer based on the Koppen-Geiger climate classification^40^ Thirty year average temperature in the watershed is 15.5 °C and annual precipitation ranges from 703 to 1,473 mm.

### Hydrologic model application

#### Model description

A physically-based, fully distributed hydrologic model, Penn State Integrated Hydrologic Model (PIHM)^4,5^ was used in this study to simulate coupled hydrologic states and fluxes. PIHM has previously been applied to simulate hydrologic process dynamics at multiple scales and in diverse hydro-climatological settings^41–45^. Processes simulated in PIHM include snowmelt, evapotranspiration (Penman–Monteith equation), interception (Rutter model), overland flow (2D diffusion wave equation), unsaturated zone infiltration (1D approximation of the Richards equation), groundwater flow (Boussinesq equation), and streamflow (1D diffusion wave equation). The model couples surface (channel routing and overland flow) and subsurface (groundwater and unsaturated flow) processes using the principle of mass conservation. Using a semi-discrete finite-volume approach, the model spatially discretizes the partial differential equations of hydrologic states into ordinary differential equations (ODEs). The system of ODEs defined on all mesh elements are assembled and solved simultaneously in time using a stiff solver based on Newton-Krylov iteration. An adaptive time-stepping scheme is used to capture the varied time scales of states.

#### Domain discretization

Triangular and linear-shaped elements in 2D, which represent land surface elements and rivers respectively, are used to discretize the model domain. Each land element is discretized into four layers: a top surface overland flow layer with variable thickness, a relatively thin unsaturated zone with a defined maximum thickness *T* (default value of *T* is 0.25 m), an intermediate unsaturated zone that extends from *T* to groundwater table, and a groundwater layer. The two lower layers have variable dimensions, as they depend on the evolving GWT depth. Each river unit is vertically discretized into two layers, with flowing river on the top and a groundwater zone below it. Use of triangular cells allow efficient and accurate representation of physiographic, climatic and hydrographic features because of their spatial adaptivity and ability to conform to sinuous boundaries^20,46,47^. Here, four discretization configurations (Fig. 1) were considered for inter-comparison: a fine discretization with 25,443 elements, a nested-gw discretization with 836 elements, a nested-wl discretization with 1,130 elements, and a coarse discretization with 752 elements. The fine discretization was generated by using a maximum area constraint of 20,000 m^2^ i.e. all Delaunay triangles in the domain were of size smaller than or equal to 20,000 m^2^. Nested discretization, which has found applications in climate^48^ and shallow flow^49–51^ modeling literature, was generated by using a maximum area constraints of 20,000 m^2^ within a polygon that subsumes the target location and 6,000,000 m^2^ outside of it. The constraining polygon used around the wetland was around 2.89 km^2^ in area, while that around the groundwater gauging station had an area of 0.65 km^2^. The coarse discretization was generated by using a maximum area constraint of 6,000,000 m^2^ in the entire watershed. All four discretizations were generated in PIHMgis^17^. The PIHMgis uses TRIANGLE mesh generator by Shewchuk^52^ to generate the constrained Delaunay triangles of a given maximum size within a defined area of the model domain.

#### Model Parameterization

Model simulation was conducted for 6 water years ranging from Oct 1^st^ 2004 to Sep 30^th^ 2010 for all four aforementioned discretizations of the domain. A water year is an annual period spanning from the start of October to September end of next year. Simulations using the four meshes were set up using PIHMgis, which facilitates automatic extraction and assignment of ecological and hydrogeological parameters, and meteorological forcings on each discretization grid. Input data to the model include descriptions of topography, soil, land cover, vegetation, geology, and meteorology. We used the 30 m resolution elevation data from National Elevation Dataset (NED)^53^, USDA-NRCS Soil Survey Geographic (SSURGO) soil data^54^, and National Land Cover Dataset (NLCD) land cover data^55^. Meteorological forcings such as precipitation, air temperature, relative humidity, wind speed, and radiation were obtained from North America Land Data Assimilation System Phase 2 (NLDAS-2) data^56^, which has a spatial and temporal resolution of 1/8 degree and an hour, respectively. Initial conditions of hydrologic states on 10/01/2004 mid-night (first hour of the simulation) were extracted from the long term simulation conducted by Liu and Kumar^27^. To map the states from the mesh configuration used in Liu and Kumar^27^ to that used here, Inverse Distance Weighted (IDW) interpolation was employed. To minimize the effects of errors introduced by IDW interpolation scheme and mismatch of mesh configuration, first year simulation was only used to let the system equilibrate with the forcings. Only simulations for the next 5 years (Oct 1^st^ 2005 to Sep 30^th^ 2010) were used for analyses. Terming years with annual precipitation in the top 20 percentile as wet and in the bottom 20 percentile as dry based on 30 years (1983–2013) precipitation data, the five year simulation period consisted of one dry year (from Oct 1^st^ 2007 to Sep 30^th^ 2008) and one wet year (Oct 1^st^ 2009 to Sep 30^th^ 2010).

All four simulations were performed using the calibration parameter set derived in Liu and Kumar^27^ (see Supplementary Table S1). Identical calibration parameters were used so as to study the isolated influence of mesh configurations on hydrologic responses, given a defined field property or spatial distribution of parameters. In Liu and Kumar^27^, the calibration was performed using two steps. The first step in the calibration process was initialization of the PIHM model with water table at the land surface. The model was then allowed to relax with no precipitation input until the stream flow recession rate matched the observed during the low flow period in summer. The modeled stream flow magnitude was then compared with the observed value. This was done because streamflow during low flow period is largely due to groundwater base flow, and hence a match between observed and modeled streamflow would indicate reasonable estimation of the groundwater distribution in summer. During this process, the hydraulic conductivity of the subsurface was calibrated uniformly across the entire model domain. Then starting from the derived groundwater table initial condition, the next step involved forcing the model with real meteorological inputs. After a one-year warm-up period, the simulation results were compared against the observed streamflow and groundwater data at the gauging station. Manual calibration of hydrogeologic parameters such as soil hydraulic conductivity, macroporosity, and soil drainage parameters, was performed in this step. Readers are encouraged to refer to Liu and Kumar^27^ for more details about the data sets and performance metrics used for calibration and validation of the model. As this study’s aim is restricted to exploring the extent to which hydrologic states simulated by a fine grid can be captured by a nested grid in the refined region, as long as identical properties are used to parameterize the four simulations, any calibration set could have been used.

### Target areas, metrics, and hydrologic variables used for intercomparison

Two target areas were considered, one for each application. The first target area was at the groundwater well location (USGS gauge #354057080362601) where daily groundwater level has been measured since 1989 (Fig. 1). The location was used to evaluate if groundwater table dynamics estimated by a fine discretization can be replicated by nested and coarse discretizations, assuming parameterization of all three discretizations was performed using identical land cover, hydrogeological, and meteorological map. If the coarse simulation did not replicate the groundwater dynamics shown by the fine simulation, but the nested simulation did, that would indicate that coarse mesh resolutions introduce errors in estimates of groundwater table dynamics. In such cases, a model user may be forced to seek alternate parameterizations in order to compensate for errors due to the use of coarse mesh resolutions. The second target area was the largest wetland (~167,000 m^2^) within the watershed (Fig. 1). The location was used to evaluate if simulated groundwater table dynamics by the fine simulation could be accurately captured by coarse and nested simulations. Groundwater table dynamics in wetlands is of interest as it is known to influence several ecohydrological functions^57^ including greenhouse gas emissions^58^, carbon and nitrogen cycles, and biodiversity^59–61^. Given that the selected wetland is small (<0.2 km^2^ in area), capturing groundwater dynamics within it requires modeling of the wetland region at fine spatio-temporal scales. As the wetland lies in the vicinity of a stream that can exchange water with it, rest of the watershed has to be modeled as well, as it is likely to influence the stage in the stream reach adjacent to the wetland. In addition, lateral flux interactions between wetland and the neighboring aquifer may also influence the groundwater dynamics within the wetland. For this application, we intercompared groundwater depth estimated by three simulations, viz., fine, nested-wl, and coarse simulation, within the wetland region. Comparisons were performed for three variables viz. average GWT, groundwater table distribution, and wet area fraction. Average groundwater table within the wetland was computed using the area-weighted method shown in equation (1).

1 $$GW{T}_{avg}=\,\frac{{\sum }_{i=1}^{i=N}GW{T}_{i}\ast Are{a}_{i}}{{\sum }_{i=1}^{i=N}Are{a}_{i}}$$

where *N* is the number of model elements lying within the National Wetland Inventory^62^ wetland boundary. Comparison of GWT table distribution was performed using exceedance probability plots that quantified the fraction of wetland elements with NSEs higher than a threshold value. NSE of the concerned variable (e.g., GWT) for coarse and nested-wl simulations was calculated w.r.t. the fine simulations. The wet area fraction (*WAF*) at any given time step was calculated using equation (2).

2 $$WAF=\frac{{\sum }_{j=1}^{j=M}Are{a}_{j}}{{\sum }_{i=1}^{i=N}Are{a}_{i}}\times 100 \% $$

where *N* is the number of elements within the wetland, and *M* is the number of wetland elements with GWT being above a prescribed threshold. Three water level thresholds viz. −0.3 m, −0.5 m, and −1.0 m were considered. The flow interactions between the wetland and its adjacent stream and aquifer were also compared given their influence on the hydrology and biochemistry of wetlands.

### Data availability

The model code and datasets generated during and/or analyzed during the current study are available from the corresponding author on request.

## Electronic supplementary material

Supplementary Information
