# Supplementary material for: Using nested discretization for a detailed yet computationally efficient simulation of local hydrology in a distributed hydrologic model
Source: Sci Rep. 2018 Apr 10;8:5785. doi: 10.1038/s41598-018-24122-7 (PMC5893575; doi:10.1038/s41598-018-24122-7)
Supplement: Supplementary file 1 — Supplementary Information [file 41598_2018_24122_MOESM1_ESM.docx]

**Using nested discretization for a detailed yet computationally efficient simulation of local hydrology in a distributed hydrologic model**

Dongdong Wang, Yanlan Liu, and Mukesh Kumar^*^

Nicholas School of the Environment

*Corresponding author

E-mail: mukesh.kumar@duke.edu

**Supplementary Information**

**Table S1 Model calibration file.**

| Parameter | Value | Parameter | Value | Parameter | Value |
| --- | --- | --- | --- | --- | --- |
| KsatH | 0.6 | Beta | 2 | Et2 | 1 |
| KsatV | 0.03 | vAreaF | 10 | rivRough | 0.2 |
| infKsatV | 0.08 | hAreaF | 1 | rivKsatH | 70 |
| macKsatH | 0.025 | VegFrac | 1 | rivKsatV | 50 |
| macKsatV | 0.02 | Albedo | 1 | rivbedThick | 1 |
| infD | 2.5 | Rough | 1.5 | rivDepth | 0.3 |
| RzD | 1.5 | Precp | 1 | rivShapeCoeff | 4 |
| macD | 2.3 | Temp | 1 |  |  |
| Porosity | 0.09 | Et0 | 1 |  |  |
| Alpha | 0.3 | Et1 | 1 |  |  |


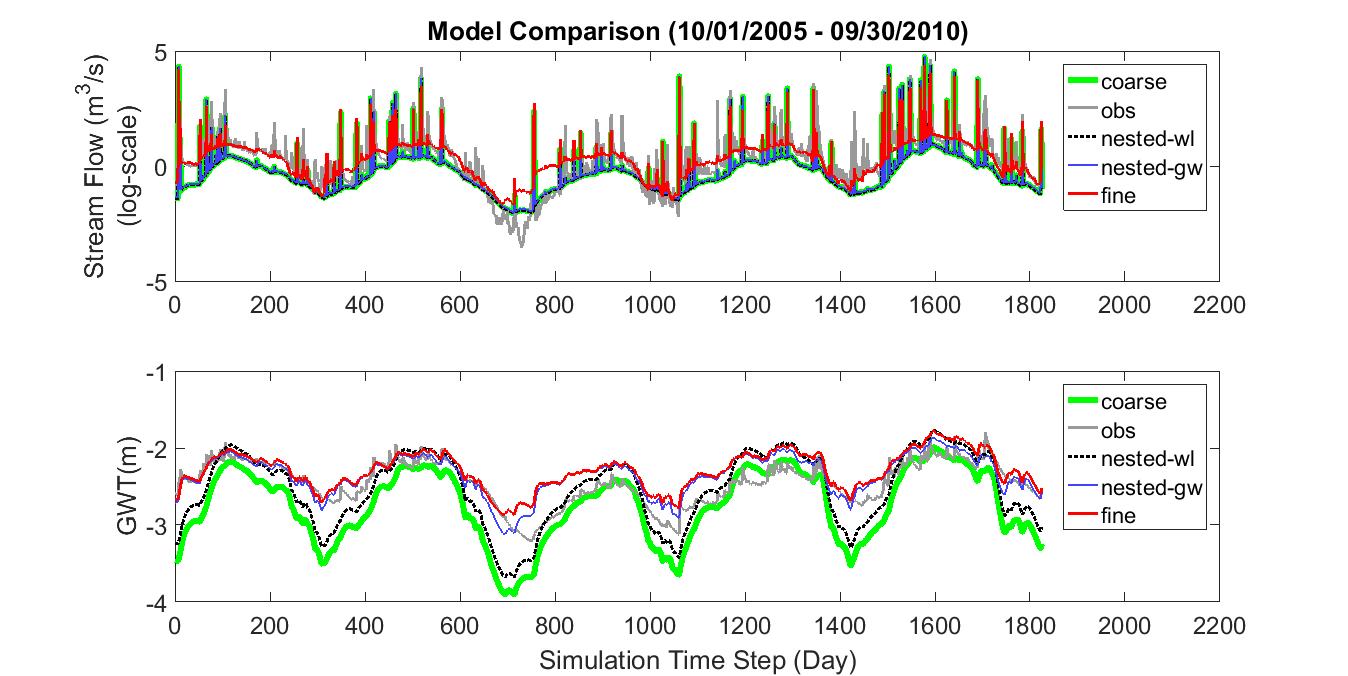


**Figure S1**. Modeled streamflow at the gauging location from fine, nested-wl, nested-gw, and coarse simulations. Log scale on the y-axis has *e* (=2.718) as its base.

**Figure S2.** Comparison of stream stage in the stream reach just upstream of the wetland.

(a)


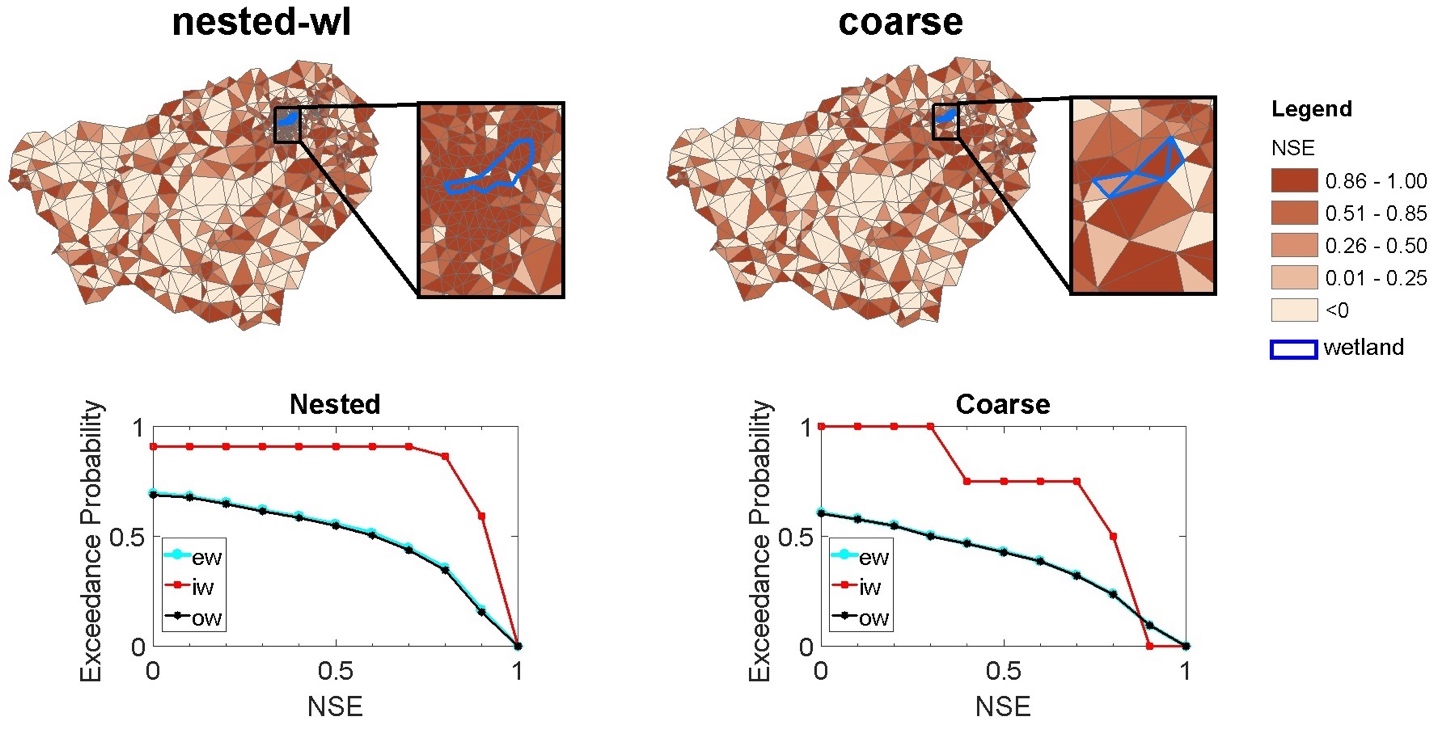


(b)


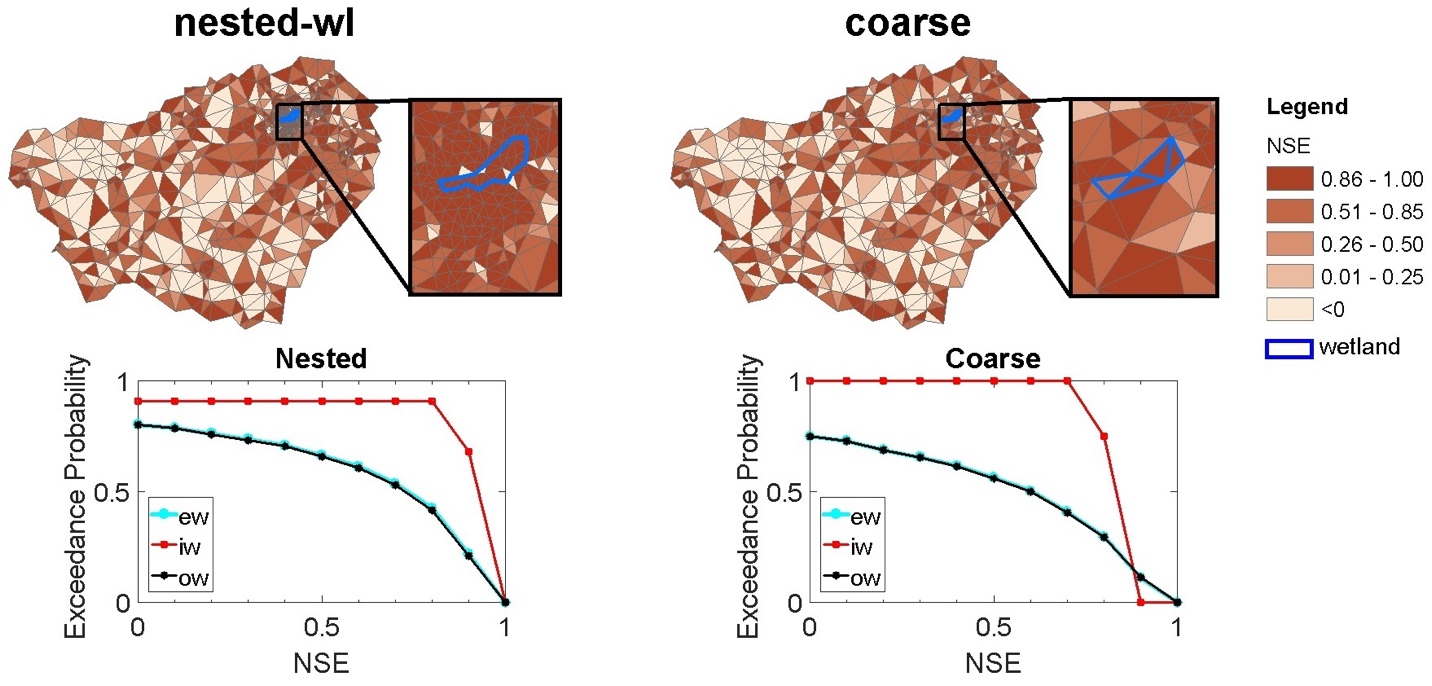


**Figure S3**. Comparison of model fit with respect to fine simulation over the 5 years simulation period. Comparison was performed using the simulated soil moisture time series in the top 25 cm of the land surface (a) and mean removed soil moisture time series. Here, ew denotes “entire watershed”, iw denotes “inside of wetland”, and ow denotes “outside of wetland”.

(a)


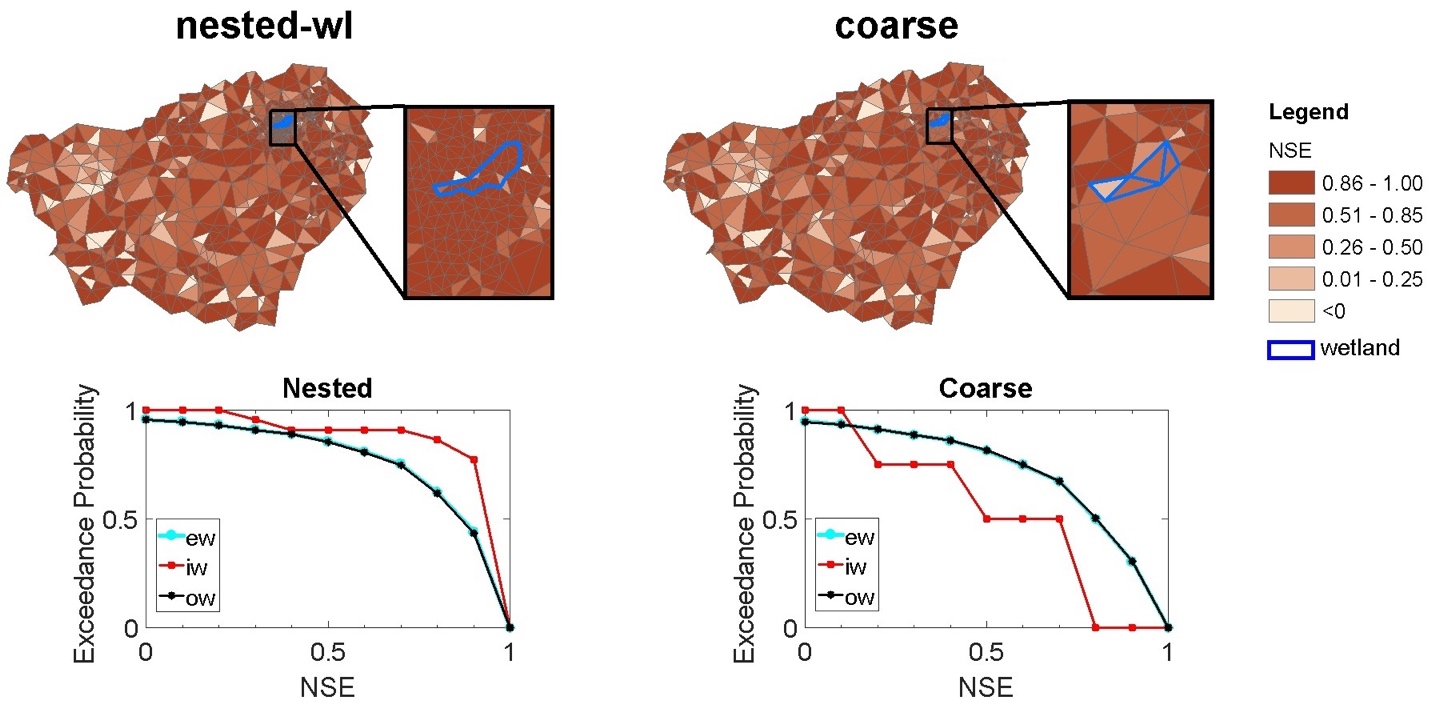


(b)


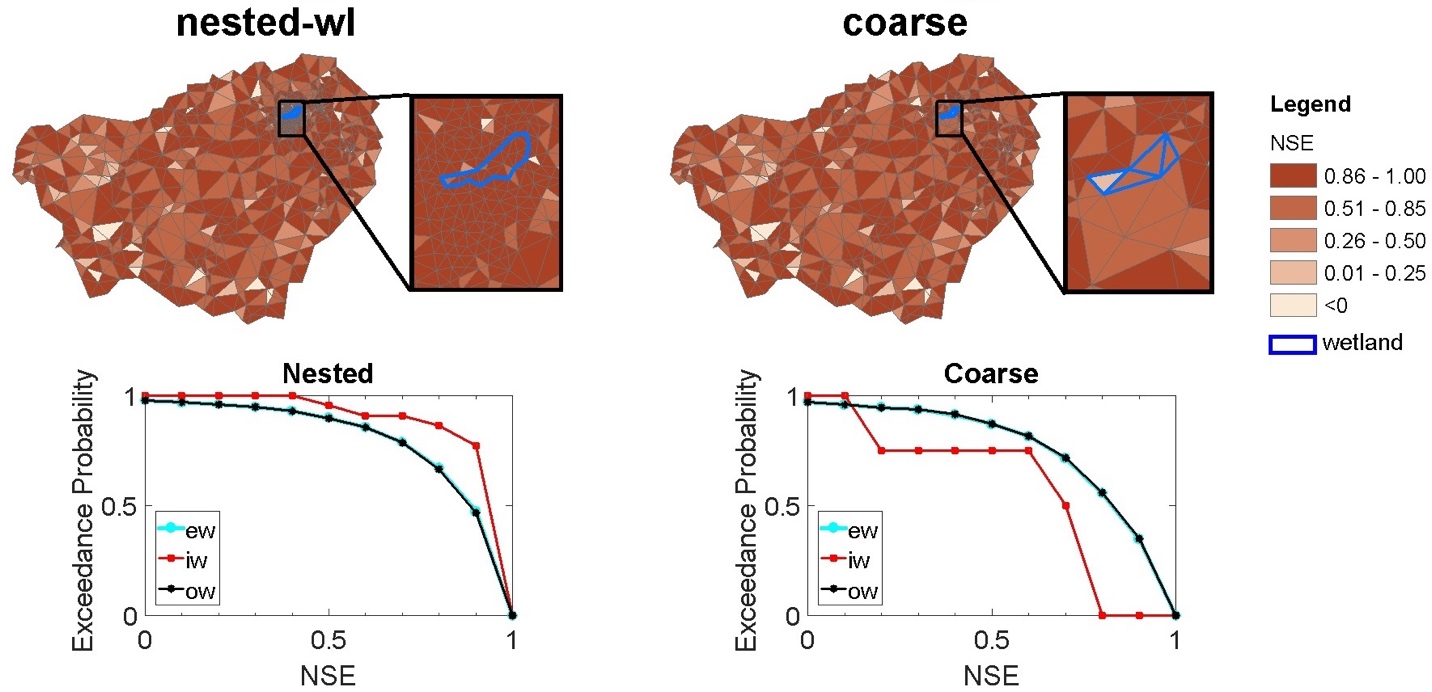


**Figure S4**. Comparison of model fit with respect to fine simulation over the 5 years simulation period. Comparison was performed using the simulated evapotranspiration time series (a) and mean removed evapotranspiration time series. Here, ew denotes “entire watershed”, iw denotes “inside of wetland”, and ow denotes “outside of wetland”.
